# Contribution of host-related immune factors to frequency and severity of infections in lung transplant recipients

**DOI:** 10.1186/s12931-026-03685-4

**Published:** 2026-05-01

**Authors:** Jiri Kufa, Petra Schneiderova, Samuel Genzor, Marketa Trajerova, Petr Jakubec, Jan Mizera, Monika Zurkova, Eva Kriegova

**Affiliations:** 1 Department of Pulmonary Diseases and Tuberculosis, Faculty of Medicine and Dentistry, Palacký University Olomouc and University Hospital Olomouc, Zdravotníku 248/7, Olomouc, 779 00 Czech Republic; 2 Department of Immunology, Faculty of Medicine and Dentistry, Palacký University Olomouc and University Hospital Olomouc, Hnevotinska 976/3, Olomouc, 779 00 Czech Republic; 3 Center for digital health, Faculty of Medicine and Dentistry, Palacký University Olomouc, Hnevotinska 976/3, Olomouc, 779 00 Czech Republic

**Keywords:** Single nucleotide polymorphisms, Cytokines, Post-transplantation infections, Infection severe risk, Blood immune profile

## Abstract

**Background:**

Lung transplantation (LTx) is associated with an increased risk of infection, causing notable morbidity and mortality at all phases post-transplantation. The contribution of host-related immune factors to susceptibility to post-LTx infections is not fully understood.

**Methods:**

The circulating blood profile, serum cytokine profiles and 28 functional single nucleotide polymorphisms in cytokine genes were investigated in 103 lung transplant recipients (LTRs) (men/women: 65/38; mean [95% confidence interval {CI}]: 5.4 [4.7–6.1] years post-LTx) using cytometry and MassARRAY genotyping; the frequency and severity of viral, bacterial and fungal infections were evaluated.

**Results:**

In total, 62 (60.2%) LTRs had frequent/severe infections requiring inpatient care (FSI) and 41 (39.8%) had infrequent infections managed in the outpatient setting (IO). We identified rs1800587 AA (*IL1A*), rs1143634 AA (*IL1B*), rs16944 GG (*IL1B*), rs1800795 CG/GG (*IL6*) and rs1800797 AG/GG (*IL6*) as being associated with susceptibility to frequent/severe infections in LTRs. A combination of ≥ 2 *IL1/IL6* risk variants was present in 53.2% of patients with frequent/severe infections compared with 17.1% with infrequent infections, increasing the likelihood of severe/frequent infections 5.5-fold (95% CI 2.17–13.6; *p* < 0.001). Moreover, patients with severe/frequent infections exhibited distinct changes in immune cell composition and cytokine profiles between 6- and 12-months post-LTx compared with others.

**Conclusion:**

Our data demonstrated that LTRs carrying *IL1/IL6* risk variants and exhibiting limited post-LTx immune reconstitution were substantially more susceptible to frequent/severe infections. These findings highlight the value of combining immunogenetic profiling with longitudinal immune monitoring to improve infection risk stratification and guide personalised post-LTx care.

**Supplementary Information:**

The online version contains supplementary material available at 10.1186/s12931-026-03685-4.

## Background

Lung transplantation (LTx) remains a life-saving therapy for individuals with end-stage lung disease, including chronic obstructive pulmonary disease, idiopathic pulmonary fibrosis, cystic fibrosis and other progressive lung diseases. Although advances in surgical techniques, perioperative care and long-term post-transplant care have substantially improved early outcomes, lung transplant recipients (LTRs) remain extremely susceptible to infectious complications [1]. The combination of chronic immunosuppression, altered airway anatomy, impaired mucociliary clearance and constant exposure of the allograft to the external environment creates favourable conditions for microbial invasion [2, 3]. Episodes of acute rejection, chronic graft dysfunction and fluctuations in immunosuppressive regimens further increase this vulnerability [4]. As a result, infection persists as one of the leading causes of long-term morbidity and mortality after LTx [5, 6], underscoring the need to better understand the risk factors associated with post-LTx infections.

Infectious complications can occur at any time after LTx and often present with atypical or subtle clinical symptoms, complicating early diagnosis. Although infections occurring early after LTx are often associated with surgical complications, airway colonisation or exposure to hospital-acquired pathogens, infections occurring more than 4 months after LTx more commonly involve opportunistic or community-acquired organisms as well as the reactivation of latent viruses and fungi [2, 3, 5, 6]. Current surveillance strategies, which rely primarily on clinical monitoring, microbiological testing and imaging methods, tend to identify infections only after symptoms appear [3, 5]. Therefore, new biomarkers are needed to identify at-risk patients, assess host-immune competence and tailor immunosuppressive therapy to individual patients.

In this study, we investigated the history of infections in a real-world cohort of LTRs and its association with host-related immune factors, such as functional genetic polymorphisms in cytokine genes, circulating immune cell status and serum cytokine levels. Understanding these immunogenetic factors may initiate individualised adjustments to immunosuppressive therapy and prophylactic regimens.

## Methods

### Patients

The study cohort consisted of 103 patients (men/women: 65/38) who underwent LTx at Motol University Hospital, Prague, Czech Republic, and were followed up at the University Hospital Olomouc, Czech Republic. The exclusion criteria were a short time after LTx (≤ 4 months) by study enrolment, short time of follow-up (≤ 12 months), and death in the first year after LTx. The received induction immunosuppression and maintenance treatment (a calcineurin inhibitor, mycophenolate mofetil and/or low-dose prednisone) protocol followed our institutional protocol, which was adapted from the Toronto Lung Transplant Program protocol [7]. The study used a mixed design, with prospective blood sample collection and retrospective collection of clinical characteristics and follow-up data.

The frequency and severity of bacterial, viral, and fungal infections were assessed in all patients. The definitions of viral, bacterial and fungal infections are based on the recommendations of the American society of transplantation [8]; chronic airway colonization was defined as persistent detection of the same microorganism in respiratory specimens over ≥ 3 months without clinical evidence of active infection. Based on the severity and frequency of infections during follow-up, patients were stratified into two groups: (1) those with infrequent infections managed in the outpatient setting (IO) and (2) those with frequent and/or severe infections requiring inpatient care, including hospitalization on a standard ward or admission to the intensive care unit (FSI). Only infections that were documented in the medical records and required medical evaluation or treatment were included in the analysis.

The study was performed in accordance with the 1964 Helsinki Declaration and its later amendments and was approved by the local Ethics Committee. Informed consent was obtained from all individuals involved in the study.

### Sample processing and flow cytometry

Peripheral blood samples were processed within 4 h after collection. Serum samples were aliquoted and frozen at -80 °C until analysis. A portion of the whole blood was used for DNA extraction and flow cytometry analysis. Only patients with paired sampling at 6 months and 1-year post-LTx and no signs of ongoing infection were included for the immunophenotyping analysis. Complex immunophenotyping of circulating immune cells and their activation markers was performed as reported elsewhere [9, 10] on a BD FACSCanto II instrument (BD Biosciences, San Jose, CA, USA). The main immune cell populations were calculated as a percentage of cell singlets. Subpopulation percentages were calculated either as a part of parental populations or percentages of all cell singlets. In all experiments, a minimum of 5,000 cells from each of the main populations was measured, and a cut-off of 500 events was used to evaluate the activation markers. Activation status was determined either by the percentage of cells expressing the selected marker (%) or as the median fluorescence intensity (MFI). For the antibodies, gating strategy and controls used see Supplementary Table S1, Figures S1 and S2. Data were analysed using FlowJo (version 10.9.0) software (BD Biosciences).

### Genetic analysis

A set of 28 functional single nucleotide polymorphisms (SNPs) in immune genes (*IL1A*: rs1800587; *IL1B*: rs16944, rs1143634; *IL1RN*: rs315952, rs579543, rs4251961; *IL1R1* rs2234650; *IL2*: rs2069762, rs2069763; *IL4*: rs2243248, rs2243250, rs2070874; *IL4R*: rs1801275; *IL6*: rs1800795, rs1800797; *CXCL8*: rs2227307, rs4073; *IL10*: rs1800871, rs1800872, rs1800896; *IL12*: rs3212227; *IL17A*: rs2275913; *IL17F*: rs763780; *TNF*: rs1800629, rs361525; *MBL2*: rs11003125, rs1800450; *IFNG*: rs2430561) was genotyped using the allele-specific MALDI-TOF MassARRAY system (Agena Bioscience, San Diego, CA, USA). The primers for polymerase chain reaction (PCR) amplification and the primer extension reaction were designed using the Agena Assay Design Suite (version 3.0) online tool and are listed in Table S2. The genotyping was performed on genomic DNA using the iPLEX Gold PCR Reagent and SpectroCHIP Kit (Agena Bioscience) in accordance with the manufacturer’s recommendations. The genotypes were obtained from MassARRAY measurements using Typer (version 5.0.9) software (Agena Bioscience).

### Analysis of cytokine serum levels

Serum levels of cytokines – interleukin (IL)-4, IL-2, CXCL10 (IP-10), IL-1β, TNF-α, CCL2 (MCP-1), IL-17 A, IL-6, IL-10, IFN-γ, IL-12p70, CXCL8 (IL-8) and active TGF-β1 – were measured using a bead-based multiplex assay (LEGENDplex Human Essential Immune Response Panel 13-plex; BioLegend, San Diego, CA, USA) according to the manufacturer’s instructions. Only patients with paired sampling at 6 months and 1-year post-LTx and no signs of ongoing infection were included for the cytokine analysis. Analysis was performed on a BD FACSCanto II flow cytometer (BD Biosciences). Data analysis was performed using the Data Analysis Software Suite for LEGENDplex (Qognit; BioLegend). Interleukin-4 levels were below the detection limit in more than 70% of samples; therefore, this analyte was excluded from further analyses.

### Statistical analysis

Statistical tests, including the chi-squared (χ²) test, odds ratio (OR), non-parametric Mann–Whitney U test, and Kruskal–Wallis test were performed using GraphPad Prism (version 8.0.1) and R statistical software (http://www.r-project.org/). The results were presented as the mean and the corresponding 95% CIs. A p-value ≤ 0.05 was considered statistically significant.

## Results

### Study population and infection outcomes

Regarding study subgroups, 41 patients (IO, 39.8%; 29 men/12 women) had infrequent infections managed in the outpatient setting and 62 patients (FSI, 60.2%; 36 men/26 women) had frequent and/or severe infections requiring inpatient care or died due to infection (12 patients, 19.4%) during the follow-up (Table 1). The mean follow-up time, defined as the period from admission to post-transplant care at University Hospital Olomouc to the last documented clinical visit, was 4.8 years (95% confidence interval [CI] 4.2–5.3 years).

**Table 1:** Demographic and clinical characteristics of the enrolled lung transplant recipients

Patient data	All LTRs *N* = 103	LTRs with IO *N* = 41	LTRs with FSI *N* = 62	*p*-value
Sex, men/women, N (%)	65/38 (63.1/36.9%)	29/12 (70.7/29.3%)	36/26 (58.1/41.9%)	0.192
Age at LTx, mean (95% CI) [years]	51.1 (48.5–53.7)	53.1 (49.4–56.7)	49.8 (46.1–53.5)	0.429
Time from LTx, mean (95% CI) [years]	5.4 (4.7–6.1)	4.7 (3.6–5.7)	5.8 (4.9–6.8)	0.128
Follow-up^#^, mean (95% CI) [years]	4.8 (4.2–5.3)	4.4 (3.6–5.3)	5.0 (4.3–5.7)	0.288
Indications for LTx: ILD/COPD/CF/other, N (%)	44/34/14/11 (42.7/33.0/13.6/10.7%)	20/15/2/4 (48.8/36.6/4.9/9.8%)	24/19/12/7 (38.7/30.6/19.4/11.3%)	0.194
FEV1 – highest value [L], mean (95% CI)	2.67 (2.51–2.83)	3.01 (2.74–3.28)	2.44 (2.25–2.63)	˂0.001
HRCT – Bronchiectasis, N (%)	28 (27.2%)	7 (17.1%)	21 (33.9%)	0.061
HRCT – Air trapping, N (%)	43 (41.7%)	16 (39.0%)	27 (43.5%)	0.649
Acute rejection: no/mild/recurrent mild/grade≥A2, N of values; N (%)	89; 34/31/11/13 38.2/34.8/12.4/14.6%)	38; 17/14/2/5 (44.7/36.8/5.3/13.2%)	51; 17/17/9/8 (33.3/33.3/17.6/15.7%)	0.306
BLAD, N (%)	42 (40.8%)	10 (24.4%)	32 (51.6%)	0.006
CLAD, N (%)	19 (18.4%)	4 (9.8%)	15 (24.2%)	0.064
CKD: no or low risk/ moderate to high risk/ very high risk*, N (%)	27/64/12 (26.2/62.1/11.7%)	11/27/3 (26.8/65.9/7.3%)	16/37/9 (25.8/59.7/14.5%)	0.533
Chronic airway colonization, N (%)	39 (37.9%)	7 (17.1%)	32 (51.6%)	˂0.001
≥ 1 for-cause bronchoscopy, N (%)	37 (35.9%)	7 (17.1%)	30 (48.4%)	0.001
Anti-HLA antibodies (DSA or non-DSA), N (%)	37 (35.9%)	16 (39.0%)	21 (33.9%)	0.594
Immunoglobulin therapy, N (%)	11 (10.7%)	0	11 (17.7%)	0.004

*Abbreviations*: *BLAD* Baseline lung allograft dysfunction, *CF* Cystic fibrosis, *CI* Confidence interval, *CKD* Chronic kidney disease, *CLAD* Chronic lung allograft dysfunction, *COPD* Chronic obstructive pulmonary disease, *DSA* Donor-specific antibodies, *FEV1* Forced expiratory volume in 1 second, *FSI* Frequent and/or severe infections in inpatient settings, *HLA* Human leukocyte antigens, *HRCT* High-resolution computed tomography, *ILD* Interstitial lung diseases, *IO* Infrequent infections managed in outpatient settings, *LTR* Lung transplant recipient, *LTx* Lung transplantation, *N* Number of patients

^#^Follow-up is defined as the period from admission to post-transplant care at the University Hospital Olomouc to the last documented clinical visit. *Prognosis of CKD by glomerular filtration rate and albuminuria categories according to KDIGO guidelines [9]

**Fig. 1:**
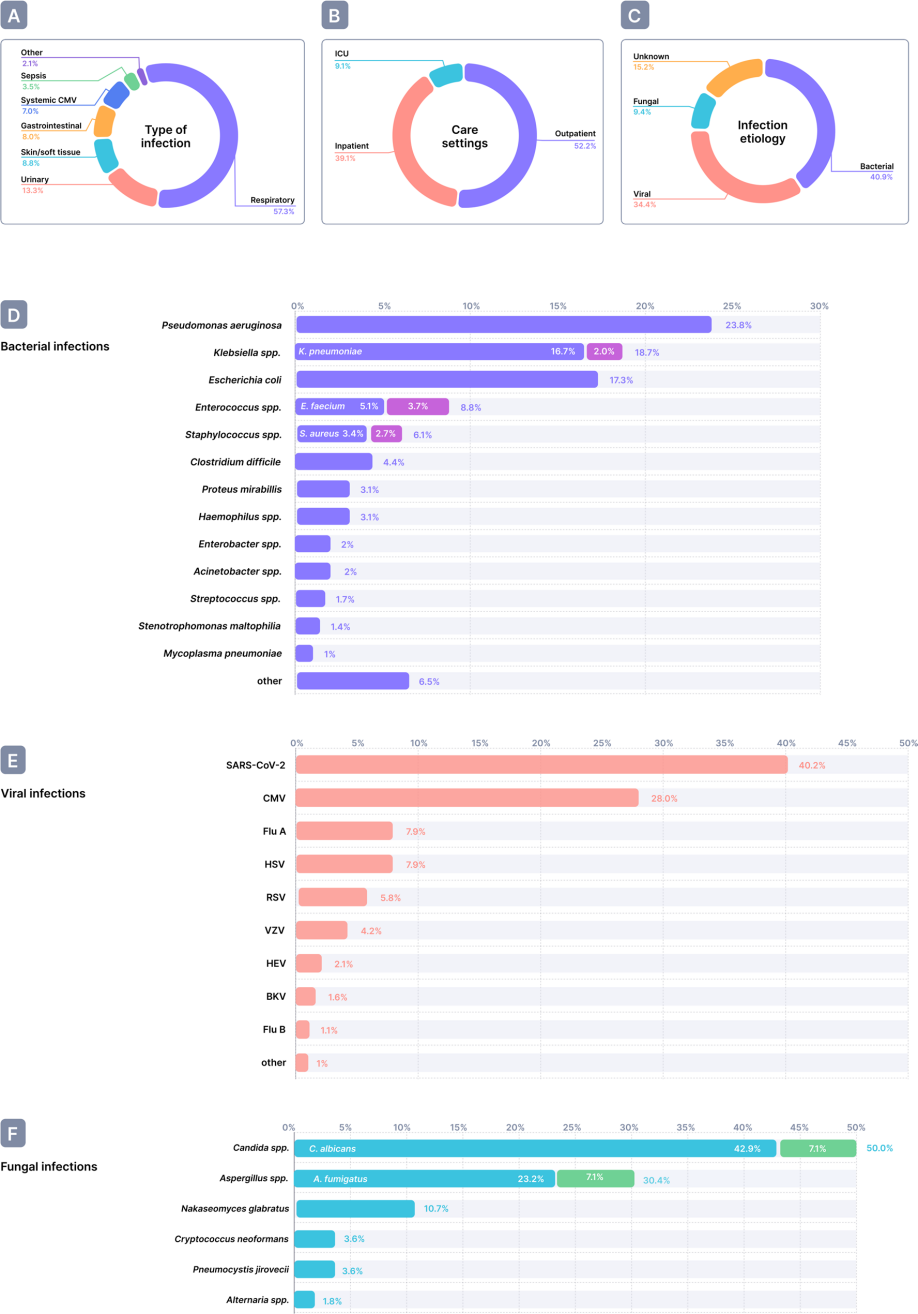
Types and etiology of infections in lung transplant recipients. The graphs depict infection types (**A**), care setting (**B**), infection etiology (**C**), and identified microbial pathogens (**D**–**F**) for all infections documented in a cohort of 103 lung transplant recipients during the entire follow-up period (mean 4.8 years, 95% CI 4.2–5.3 years). Only infections occurring more than 4 months post-transplantation were included. For bacterial and fungal species (panels D and F), both the overall proportion of each species and the most prevalent representative of that species are shown separately, with the remaining representatives depicted in a different color. *Nakaseomyces glabratus* was previously known as *Candida glabrata* (reclassified in 2022). Abbreviations: BKV, BK virus; CMV, Cytomegalovirus; Flu A, Influenza A virus; Flu B, Influenza B virus; HEV, Hepatitis E virus; HSV, Herpes simplex virus; ICU, intensive care unit; RSV, Respiratory syncytial virus; VZV, Varicella-zoster virus

Detailed demographic and clinical characteristics, including the indications for LTx, are provided in Table 1. Information on the etiological agents, the affected organ systems and number of bronchoscopies is provided in Supplementary Table S3 and Fig. 1. Among the detected infections, 40.9% were bacterial, 34.4% were viral, 9.4% were fungal, and 15.2% were of unknown etiology (Fig. 1, Table S3). Of the 62 patients classified in the frequent/severe infection group, only three patients were included solely due to hospitalisation for COVID-19. In addition, 37.9% of patients had chronic airway colonisation, which was less common in patients with infrequent versus frequent/severe infections (17.1% vs. 51.6%; *p* < 0.001). Patients with infrequent infections also underwent fewer for-cause (non-surveillance) bronchoscopies than patients with frequent/severe infections (17.1% vs. 48.4%; *p* = 0.001). However, the prevalence of chronic airway colonization did not differ between patients who did and did not undergo for-cause bronchoscopies (*p* = 0.091). The distribution of patients with cystic fibrosis among individuals with chronic airway colonization did not differ between the studied infection groups, indicating that this factor is unlikely to explain the observed association with infection severity. There were no significant differences between patients with infrequent infections and those with frequent/severe infections in terms of sex, age, transplant indication or the prevalence of chronic kidney disease. Similarly, no differences were observed in the incidence of acute rejection, chronic lung allograft dysfunction, high-resolution computed tomography findings (bronchiectasis, air trapping) or the presence of anti-human leukocyte antigen (HLA) antibodies. Patients with frequent/severe infections were more likely to have baseline lung allograft dysfunction (51.6% vs. 24.4%; *p* = 0.006) as well as a lower personal best absolute forced expiratory volume in 1 s (2.44 vs. 3.01 L; *p* < 0.001) than patients with infrequent infections.

### Impact of cytokine gene polymorphisms on infection frequency and severity in lung transplant recipients

**Fig. 2:**
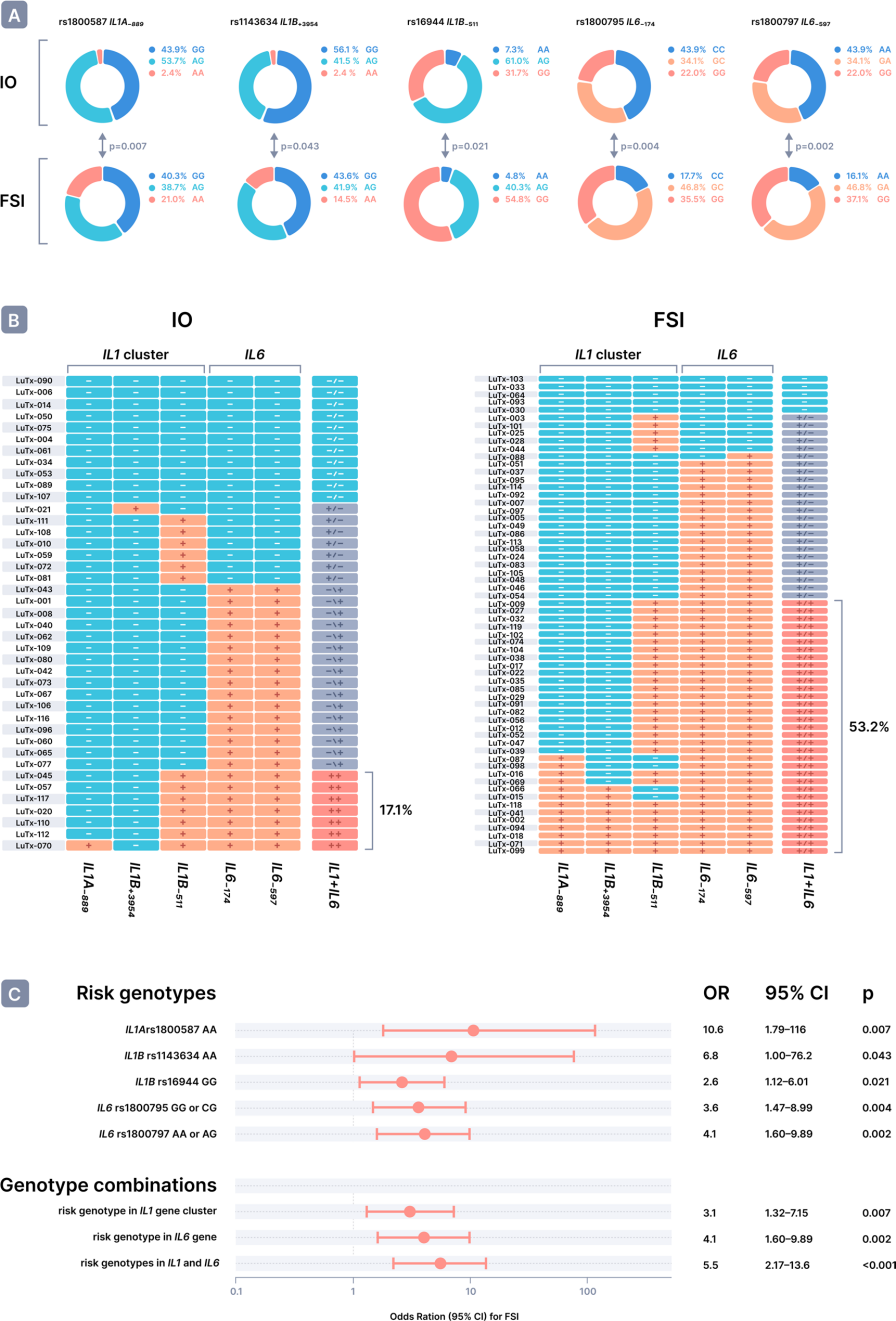
Association of interleukin gene polymorphisms with infection frequency and severity in lung transplant recipients. **A** Distribution of rs1800587 (*IL1A*_–889_), rs1143634 (*IL1B*_+3954_), rs16944 (*IL1B*_–511_), rs1800795 (*IL6*_–174_) and rs1800797 (*IL6*_–597_) genotypes in subgroups of lung transplant recipients (LTRs, *n* = 103), divided according to the frequency and severity of infections as follows: patients with infrequent infections managed in outpatient settings (IO; *n* = 41), and patients frequent/severe infections in inpatient settings (FSI; *n* = 62). Risk genotypes associated with an increased risk of frequent/severe infections are highlighted in red and orange. *P* values ≤ 0.05 are considered significant. **B** Diagram showing combinations of risk genotypes carried by individual LTRs. Presence of a given risk genotype is indicated by “+” (orange), and non-risk genotypes by “−” (blue). The last column indicates concurrent carriage of genotypes within the *IL1* cluster (*IL1A*, *IL1B*) and *IL6*; carriage of a risk genotype in at least one *IL1* cluster gene together with a risk genotype in *IL6* is denoted as “++” (red). **C** Odds ratios (ORs) for the association between carriage of risk genotypes and infection frequency/severity in LTRs. OR > 1 indicates a higher prevalence of the genotype(s) in the frequent/severe infection group. ORs with 95% confidence intervals (CIs) are shown; p values ≤ 0.05 are considered significant

Among the studied functional SNPs, five SNPs in the *IL1A*,* IL1B* and *IL6* genes (*IL1A*_− 889_ rs1800587, *IL1B*_+ 3954_ rs1143634, *IL1B*_− 511_ rs16944, *IL6*_− 174_ rs1800795 and *IL6*_− 597_ rs1800797) were associated with frequent/severe infections (χ² test; Fig. 2, Tables S4 and S5). Specifically, the *IL1A*_− 889_ AA genotype (21.0% vs. 2.4%, OR 10.6, 95% CI 1.79–116, *p* = 0.007), *IL1B*_+ 3954_ AA genotype (14.5% vs. 2.4%, OR 6.79, 95% CI 1.00–76.2, *p* = 0.043) and *IL1B*_− 511_ GG genotype (54.8% vs. 31.7%, OR 2.62, 95% CI 1.12–6.01, *p* = 0.021) were more common in patients with frequent/severe infections (Fig. 2, Table S4). In addition, of the patients with frequent/severe infections, 61.3% carried at least one *IL1* risk genotype compared with 34.1% of those with infrequent infections (OR 3.05, 95% CI 1.32–7.15, *p* = 0.007) (Fig. 2). Regarding *IL6* SNPs, patients with frequent/severe infection were more likely to be carriers of the rs1800795 G allele (CG and GG genotypes) or rs1800797 G allele (AG and GG genotypes) than those in the infrequent infection group (82.3% vs. 56.1%, OR 3.63, 95% CI 1.47–8.99, *p* = 0.004, and 83.9% vs. 56.1%, OR 4.07, 95% CI 1.60–9.89, *p* = 0.002, respectively) (Table S4). These *IL6* SNPs were in linkage disequilibrium; the rs1800795–rs1800797 GG and CA haplotypes were the most common (50.5% and 48.6%, respectively), and only two individuals in our cohort carried the rare CG haplotype (0.9%) (Fig. 2).

### Impact of combinations of risk genotypes on infection frequency and severity in lung transplant recipients

When analysing combinations of risk genotypes, patients carrying *IL1* risk genotype/s together with *IL6* risk genotype/s were markedly overrepresented in the frequent/severe infection group (53.2% vs. 17.1%) and showed a significantly increased likelihood of infection (OR 5.53, 95% CI 2.17–13.62, *p* < 0.001) compared with those in the infrequent infection group (Fig. 2). For a comparison of risk genotype frequencies between LTRs and the general population, as well as for the distribution of risk genotypes across different world populations, see Tables S6 and S7. Overall, these findings suggest that functional polymorphisms in the *IL1* and *IL6* genes are associated with an increased susceptibility to frequent/severe infections in LTRs. The risk appears to be particularly pronounced in patients carrying combined *IL1* and *IL6* risk genotypes.

### Circulating immune cells and their association with infection risk

**Fig. 3:**
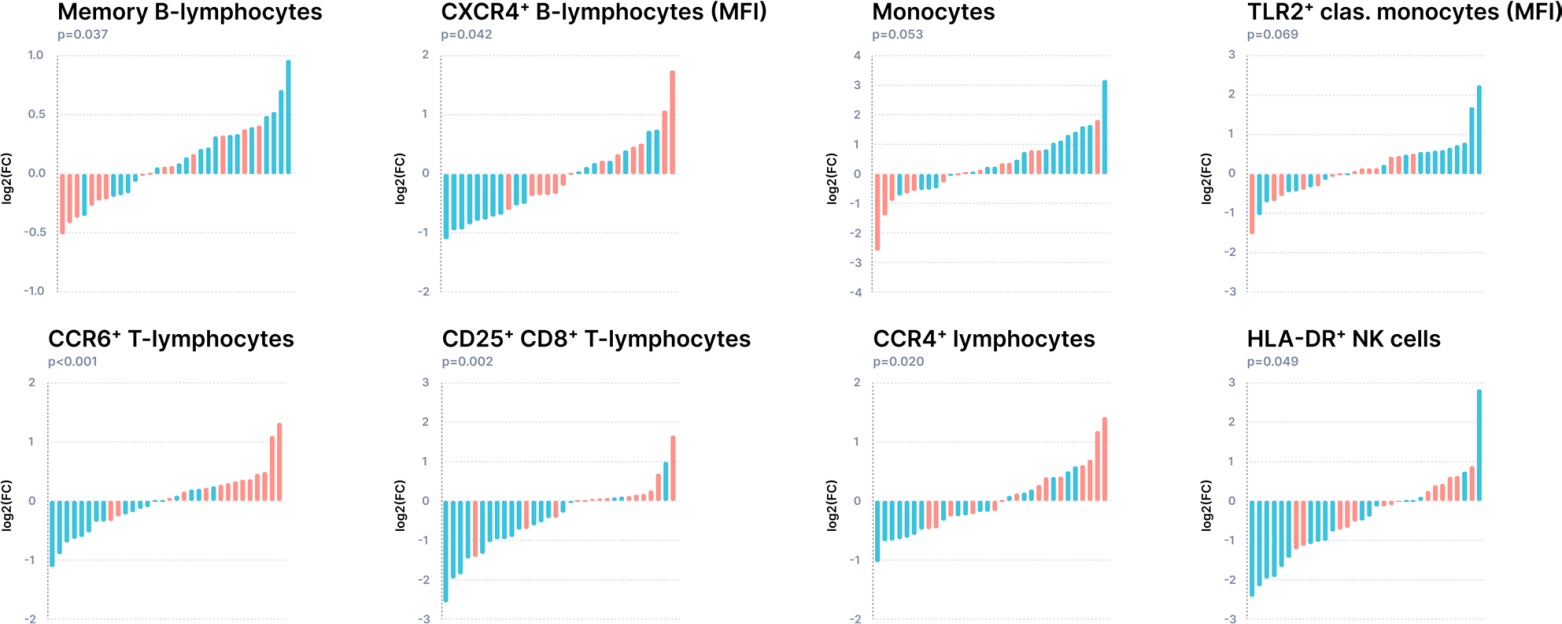
Analysis of immune cell populations in paired samples from lung transplant recipients. Paired blood samples were collected at an average of 6 months and 1 year after lung transplantation (mean 5.7 months, 95% [confidence interval] CI 5.01–6.3 months, and 14.3 months, 95% CI 12.5–16.1 months, respectively). Patients had no signs of current infection at the time of blood collection. Differences in immune cell proportions between paired samples are presented as log₂ fold change (FC). Blue bars represent FCs in patients with infrequent infections (≤2/year) managed in outpatient settings (IO; *n* = 18), and red bars represent FCs in patients with frequent/severe infections in inpatient settings (FSI; *n* = 14). Between-group differences (IO vs. FSI) are shown as *p* values; *p* ≤ 0.05 are considered significant. For more cellular populations see Supplementary data

Next, we analysed circulating immune cells and their main activation markers in 32 patients who had paired samplings at 6 months (mean 5.7 months, 95% CI 5.0–6.3) and 1-year post-LTx (mean 14.3 months, 95% CI 12.5–16.1 months); all samples were taken at a time when the patients had no signs of ongoing infection. Subanalysis revealed differences in fold change (FC) between patients with infrequent infections (*n* = 18) and those with severe/recurrent infections (*n* = 14) in the increase in memory B cells (1.19 vs. 0.99; *p* = 0.037), monocytes (2.04 vs. 1.14; *p* = 0.053) and TLR2 expression on classical monocytes (1.49 vs. 0.98; *p* = 0.069) and decrease in CCR6⁺ T cells (0.85 vs. 1.33; *p* < 0.001), CD25⁺CD8⁺ T cells (mean 0.67 vs. 1.16; *p* = 0.002), CCR4⁺ T cells (0.90 vs. 1.29; *p* = 0.020), CXCR4 expression in B cells (0.88 vs. 1.26; *p* = 0.042) and HLA-DR⁺ NK cells (0.99 vs. 1.03; *p* = 0.049) (Figs. 3 and S3). Due to the relatively small cohort size, multivariable modelling adjusting for potential confounders (e.g., age or underlying diagnosis) was not performed to avoid model overfitting. Consequently, the observed associations should be interpreted as exploratory and require validation in larger cohorts. Overall, these findings indicate distinct longitudinal immune cell dynamics between patients with frequent/severe infections and infrequent infections.

### Dynamics of serum cytokine levels after lung transplantation and their association with infection

**Fig. 4:**
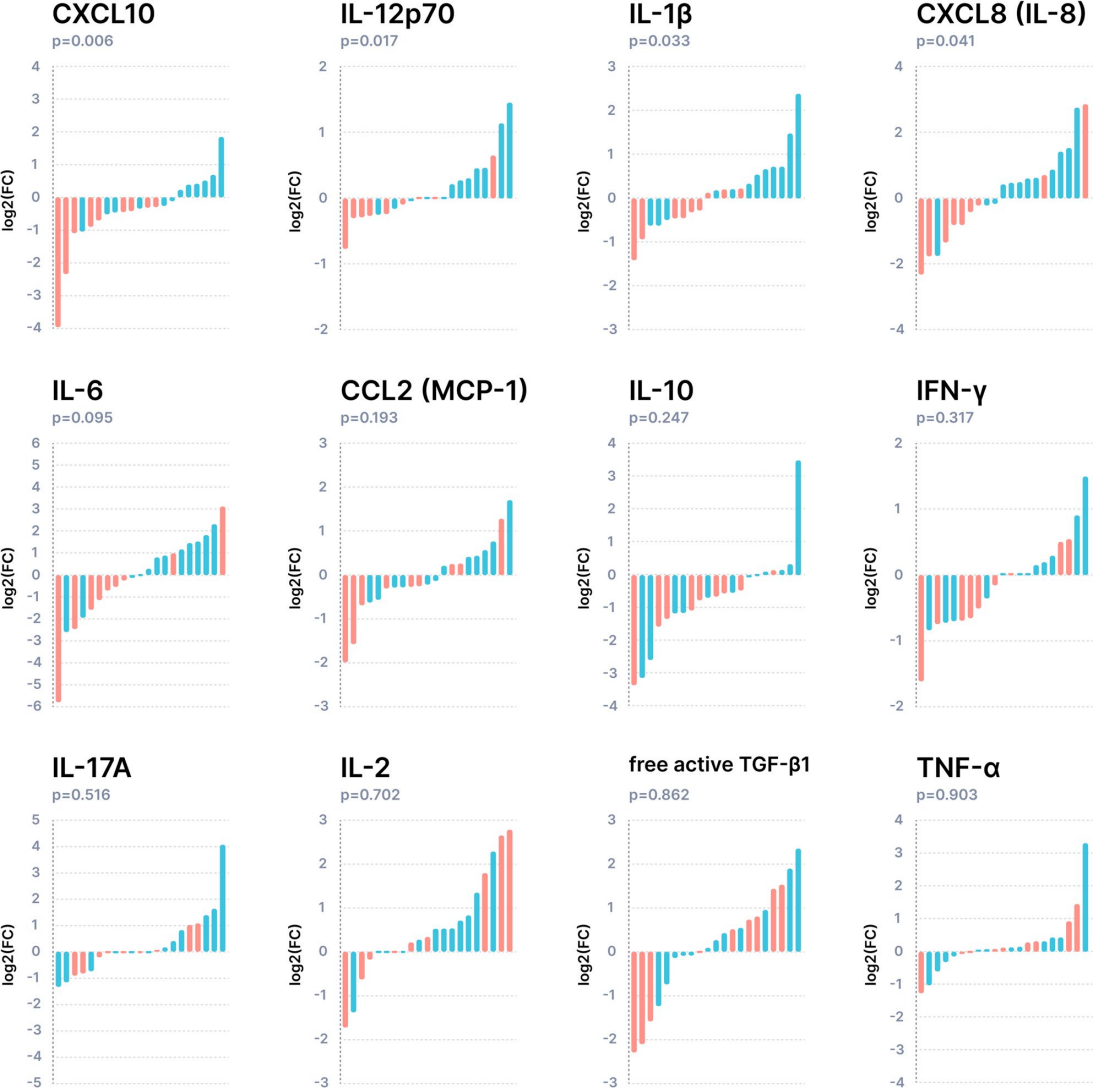
Analysis of cytokine serum levels in paired samples from lung transplant recipients. Cytokine levels were measured in paired serum samples taken 6 months (median; mean 6.1, 95% confidence interval [CI] 5.5–6.7 months) and 12 months (median; mean 12.9, 95% CI 11.5–14.3 months) after lung transplantation. Changes in cytokine levels in paired samples are presented as log₂ fold change (FC). Blue bars represent FCs in patients with infrequent infections (≤2/year) managed in outpatient settings (IO; *n* = 12), and red bars represent FCs in patients with frequent/severe infections in inpatient settings (FSI; *n* = 9). log₂FC > 0 represents an increase and log₂FC < 0 represents a decrease in cytokine level over time. Between-group differences (IO vs. FSI) are shown as *p* values; *p* ≤ 0.05 are considered significant

We assessed cytokine levels in 47 patients sampled approximately 1 year post-LTx (mean 13.7 months, 95% CI 12.5–14.8 months) at a time when they had no signs of ongoing infection in the samplings; 21 patients provided a paired sample at 6 months (mean 6.1 months, 95% CI 5.5–6.6) post-LTx. The serum levels of the analysed cytokines (IL-2, CXCL10, IL-1β, TNF-α, CCL2, IL-17 A, IL-6, IL-10, IFN-γ, IL-12p70, CXCL8 [IL-8], free active TGF-β1) showed huge interindividual variability (Figure S4). In patients with infrequent infections, the serum levels of most cytokines (11 of the 12 analysed) exhibited the same levels or an increase (overall median FC ≥ 1) between 6 months and 1-year post-LTx, with only IL-10 showing a decrease (FC < 1) (Figure S5). In a subgroup of patients with severe/frequent infections, the majority of cytokines showed a decrease, with only three (free active TGF-β1, IL-2 and TNF-α) elevated between 6 months and 1-year post-LTx (Figure S5). Regarding individual cytokines, the overall median FC of individual cytokines was higher in patients with infrequent infections than in patients with severe/frequent infections (mean 1.21 vs. 0.84, *p* = 0.006) (Figure S5). In particular, FCs in IL-1β, CXCL10 (IP-10), IL-8 and IL-12 were higher in patients with infrequent infections than in those with severe/frequent infections (*p* ≤ 0.05) (Fig. 4). In terms of serum cytokine levels (expressed as delta), between 6 and 12 months, patients with infrequent infections showed an elevation in IL-1β, CXCL10 (IP-10), IFN-γ, IL-6, IL-8 and IL-12 compared with those with severe/frequent infections (*p* ≤ 0.05) (Fig. 4). Multivariable modelling was not performed due to the limited cohort size to avoid model overfitting; therefore, the findings should be considered exploratory and require validation in larger cohorts. No patient included in the cytokine analysis had detectable donor-specific antibodies (DSA) at the time of sampling. In addition, the proportion of patients with acute cellular rejection (ACR) did not differ between the studied groups at either 6- or 12-months post-transplantation, suggesting that these factors are unlikely to have influenced the cytokine measurements. Overall, these findings suggest a reduced longitudinal cytokine serum levels in patients with frequent/severe infections compared with those with infrequent infections.

## Discussion

Lung transplant recipients are highly vulnerable to infectious complications, which are a major factor contributing to long-term morbidity and mortality following LTx. In this study, we investigated the contribution of host-related immune factors to susceptibility to post-LTx infections and revealed that genetic polymorphisms in cytokine genes and immune system status were associated with the frequency and severity of infections in LTRs.

Lung transplant recipients typically exhibit higher infection rates than recipients of other solid organs due to the significant immunosuppressive therapy requirements as well as the unique challenges posed by lung anatomy and function [2, 3]. Most studies in LTRs have focused on the prevalence of early infections [5, 12, 13], but little is known about infections that occur later than 4 months after LTx. In our cohort of LTRs, frequent/severe infections occurred in 2/3 of our patients, with only 1/3 having infrequent infections. Among infections, bacterial infections were the most common (41%), followed by viral infections, including SARS-CoV-2 (34%), and fungal infections (9%); in 15% of cases, the etiology of infection remained unknown. Although identifying patients at high risk of infection can facilitate the implementation of effective anti-infective prophylaxis and the optimisation of immunosuppression after LTx, there is only limited data on the contribution of host risk factors to infection frequency and severity after LTx. This is particularly important because of the often-atypical clinical course of infections in LTRs, leading to delayed diagnosis and increased mortality. Although SARS-CoV-2 infection was common in this cohort during the pandemic period, it did not substantially influence the classification of patients into the frequent/severe infection group, as only a minority of cases were attributable solely to COVID-19 related hospitalisation.

Based on the reported associations between sepsis/infections and cytokine polymorphisms, we have questioned whether functional polymorphisms in cytokine genes associated with high and low cytokine production may contribute to the increased frequency/severity of infections in LTRs. Among the tested SNPs, the polymorphisms in *IL1* and *IL6* genes assumed prominence in terms of contributing to susceptibility to infections in LTRs. Specifically, we identified that rs1800587 AA (*IL1A*), rs1143634 AA (*IL1B*), rs16944 GG (*IL1B*), rs1800795 CG/GG (*IL6*) and rs1800797 AG/GG (*IL6*) were associated with susceptibility to infections in LTRs. The *IL1A*,* IL1B* and *IL6* genes code for key inflammatory mediators – IL-1α, IL-1β and IL-6 – implicated in the host’s immune response to infections and progression to sepsis [14–16]. The *IL1A* and *IL1B* genes encode pro-inflammatory cytokines playing a pivotal role in innate immunity and inflammation against microbes as well as in conditions of tissue injury associated with infections and sterile conditions [14]. Functional gene polymorphisms in the *IL1* gene cluster leading to altered levels of IL-1α and IL-1β production or activity have been associated with susceptibility to infections and the severity of inflammatory responses, as demonstrated for bacteria [17–20], COVID-19 [10] and fungi [21]. Among patients with frequent/severe infections, 61.3% carried at least one *IL1* risk genotype compared with 34.1% of those with infrequent infections. The *IL1* risk genotype enhanced the likelihood of infection in LTRs by more than three times, similar to reports on kidney transplant recipients [18, 19]. Notably, only homozygous carriers of rs1800587 AA (*IL1A*), rs1143634 AA (*IL1B*) and/or rs16944 GG (*IL1B*) had a higher likelihood of infection after LTx than noncarriers. Polymorphisms in the *IL6* gene, which encodes one of the earliest inflammatory mediators released during infection, have also been associated with the risk of infection [22], and there is evidence that IL-6 is valuable for the early diagnosis and assessment of sepsis [23]. In our cohort, a combination of *IL1* and *IL6* risk variants was present in 53.2% of patients with frequent/severe infections compared with only 17.1% of those with infrequent infections, representing a 5.5-fold increase in the likelihood of developing severe infections after LTx. Interestingly, the frequency of *IL6* risk alleles in the group with frequent/severe infections was similar to that in our population, whereas in patients with infrequent infections, these risk alleles were underrepresented. By contrast, risk genotypes in *IL1* genes were significantly overrepresented in patients with frequent/severe infections, whereas their frequency in those with infrequent infections was similar to the frequency of these rare genotypes in our population.

Next, we investigated immune cell dysfunction in LTRs, as it plays a pivotal role in infection and sepsis pathogenesis. Indeed, patients with frequent/severe infections exhibited markedly attenuated changes in circulating immune cell composition between 6 and 12 months post-LTx, whereas the infrequent infection group demonstrated improved immune fitness, as shown by the increase in memory B cells and monocytes and their TLR2 expression, decreases in CD25⁺CD8⁺ T cells and CCR4⁺ and CCR6⁺ T cells and an increase in the spectrum of pro-inflammatory cytokines evident in follow-up samples. The key role of B cells in sepsis has also been reported by others, where the lowering of early B lymphocyte subsets, including memory B cells, was associated with higher sepsis-related mortality and had a predictive value for sepsis [24–26]. Moreover, low monocyte expression may predispose to infections, as these immune effector cells protect against microbial pathogens and promote immune surveillance [27]. In addition, the elevation of CCR6^+^ T cells, particularly Th17 cells and Tregs, has been reported in sepsis/severe infection while contributing to immune cell recruitment to infection sites and the modulation of inflammation [28]. Regarding NK cells, a population playing a protective role from viral infections [29], patients with infrequent infections show a reduced proportion of HLA-DR^+^ NK cells in the 6- to 12-month period post-transplant; this was not observed in the group with frequent/severe infections. Whether the decrease in activated NK cells in the group with infrequent infections may be related to less stimulation by chronic airway colonisation, which was less frequent than in the group with frequent/severe infections, deserves further research. In addition, patients with infrequent infections, but not those with frequent/severe infections, exhibited an increase in serum levels of pro-inflammatory cytokines IL-1β, CXCL10, IFN-γ, IL-6, IL-8 and IL-12 in the 6- to 12-month period post-transplant. The lack of direct correlation between cytokine gene polymorphisms, baseline serum cytokine levels, and peripheral blood immune subpopulations is not unexpected. Functional polymorphisms in cytokine genes are more likely to affect local immune responses at the site of infection, whereas the cytokine and cellular analyses in this study were performed under clinically stable conditions without active infection, and therefore may not reflect infection-driven immune activation.

This study has several limitations. Only a modest group of patients was examined, but the study is still one of the largest of its kind to analyse host-immune factors in LTx. We did not analyse the dosage of immunosuppressive medication, underlying comorbidities, age, the type of infections or the affected organ systems as possible confounding factors. Moreover, we did not evaluate mild infections (respiratory, gynaecological), and susceptibility to infection was viewed separately from that of pathogen genetics. We are also aware that other genetic factors may play a role in other populations. Moreover, further studies are warranted to clarify the role of recurrent infections, early CMV disease, individual modifications of immunosuppressive therapy, and primary diagnosis in the longitudinal evolution of infection frequency and severity in LTRs. This study should be considered exploratory and hypothesis-generating due to the limited cohort size.

## Conclusions

In summary, our findings demonstrate that frequent/severe infectious complications in LTRs occur more frequently in genetically predisposed patients with *IL1/IL6* risk alleles. Furthermore, changes in the representation of circulating immune cell subsets and cytokine profiles distinguish patients prone to frequent/severe infections from patients with infrequent infections. Despite the modest size of the study cohort, these results underscore the importance of considering host-immune factors in LTx care. Such approaches promise earlier identification of patients at high risk of infection who could benefit from less potent immunosuppression and more intensive preventive measures, ultimately leading to a reduction in the burden of frequent/severe infections in LTRs.

## Supplementary Material

Supplementary Material 1.

Supplementary Material 2.

## Data Availability

The data that support the findings of this study are not openly available due to reasons of sensitivity and are available from the corresponding author upon reasonable request. Data are located in controlled access data storage at University Hospital Olomouc, Olomouc, Czech Republic.

## Abbreviations

BLAD: Baseline lung allograft dysfunction
CI: Confidence interval
CKD: Chronic kidney disease
CLAD: Chronic lung allograft dysfunction
CMV: Cytomegalovirus
COPD: Chronic obstructive pulmonary disease
DSA: Donor-specific antibodies
FC: Fold change
FEV1: Forced expiratory volume in 1 second
FSI: Frequent and/or severe infections in inpatient settings
HRCT: High-resolution computed tomography
ICU: Intensive care unit
IL: Interleukin
ILD: Interstitial lung diseases
IO: Infrequent infections managed in outpatient settings
LTR: Lung transplant recipient
LTx: Lung transplantation
MFI: Median fluorescence intensity
N: Number
OR: Odds ratio
SNP: Single nucleotide polymorphism
