# Supplementary material for: Contribution of host-related immune factors to frequency and severity of infections in lung transplant recipients
Source: Respir Res. 2026 May 1;27:256. doi: 10.1186/s12931-026-03685-4 (PMC13289490; doi:10.1186/s12931-026-03685-4)
Supplement: Supplementary file 2 — Supplementary Material 2. [file 12931_2026_3685_MOESM2_ESM.docx]

**supplementarY tables anD figures**

**Contribution of host-related immune factors to frequency and severity of infections in lung transplant recipients** (Kufa J, Schneiderova P, Genzor S, Jakubec P, Mizera J, Trajerova M, Zurkova M, Kriegova E)

**Table S1. List of antibodies used for flow cytometry analysis of circulating immune cells.**

| **Marker** | **Clone** | **Fluorophore** | **Cat. No.** |  | **Vendor** | **RRID_ID** |
| --- | --- | --- | --- | --- | --- | --- |
| CD11b | APC | CBRM1/5 | 301410 |  | BioLegend | AB_2280647 |
| CD27 | APC | M-T271 | 356410 |  | BioLegend | AB_2561957 |
| CD45RO | APC | UCHL1 | 304210 |  | BioLegend | AB_314426 |
| CD49d | APC | 9F10 | 304308 |  | BioLegend | AB_2130041 |
| CD69 | APC | FN50 | 310910 |  | BioLegend | AB_314845 |
| TLR4 | APC | HTA125 | 312816 |  | BioLegend | AB_2562487 |
| CD14 | APC-Cy7 | HCD14 | 325620 |  | BioLegend | AB_830693 |
| CD19 | APC-Cy7 | SJ25C1 | 363010 |  | BioLegend | AB_2564193 |
| CD4 | APC-Cy7 | RPA-T4 | 300518 |  | BioLegend | AB_314086 |
| CD25 | BV421 | M-A251 | 356114 |  | BioLegend | AB_2562164 |
| CD64 | BV421 | 10.1 | 305020 |  | BioLegend | AB_2561828 |
| CTLA-4 | BV421 | BNI3 | 369606 |  | BioLegend | AB_2616795 |
| CXCR4 | BV421 | 12G5 | 306518 |  | BioLegend | AB_11146018 |
| CCR7 | BV510 | G043H7 | 353232 |  | BioLegend | AB_2563866 |
| CD15 | BV510 | W6D3 | 563141 |  | BD | AB_2738025 |
| HLA-DR | BV510 | G46-6 | 563083 |  | BD | AB_2737994 |
| CD3/CD16+CD56 | FITC/PE | UCHT1/3G8+MEM-188 | 319101 |  | BioLegend | AB_314999 |
| CD54 | FITC | HA58 | 353108 |  | BioLegend | AB_10900254 |
| CCR4 | PE | L291H4 | 359412 |  | BioLegend | AB_2562433 |
| CCR6 | PE | G034E3 | 353410 |  | BioLegend | AB_10913815 |
| CD16 | PE | 3G8 | 302008 |  | BioLegend | AB_314208 |
| TLR2 | PE | 11G7 | 565349 |  | BD | AB_2739202 |
| CD16 | PE-Cy7 | 3G8 | 302016 |  | BioLegend | AB_314216 |
| CD8 | PE-Cy7 | SK1 | 344712 |  | BioLegend | AB_2044008 |
| CXCR2 | PE-Cy7 | 5E8/CXCR2 | 320716 |  | BioLegend | AB_2564597 |
| PD-1 | PE-Cy7 | EH12.2H7 | 329918 |  | BioLegend | AB_2159324 |
| TCR Vα24-Jα18 | PE-Cy7 | 6B11 | 342912 |  | BioLegend | AB_2562230 |
| CD127 | PerCP-Cy5.5 | A019D5 | 351322 |  | BioLegend | AB_10897104 |
| CD3 | PerCP-Cy5.5 | SK7 | 332771 |  | BD | AB_2868620 |
| CXCR1 | PerCP-Cy5.5 | 8F1/CXCR1 | 320622 |  | BioLegend | AB_2565552 |
| CXCR3 | PerCP-Cy5.5 | G025H7 | 353714 |  | BioLegend | AB_10962908 |

**Table S2. Primers used in polymerase chain reaction amplification and primer extension on the Agena Bioscience platform.**

| **SNP** | **Gene (SNP position)** | **Alleles** | **Forward primer sequence (5’ to 3’)**  **Reverse primer sequence (5’ to 3’)** | **Extension primer sequence (5’ to 3’)** | **Well** |
| --- | --- | --- | --- | --- | --- |
| **rs1800587** | *IL1A* -889 | G/A | ACGTTGGATGGGCTGGCCACAGGAATTATA  ACGTTGGATGGGAAGGCATGGATTTTTAC | tacTAATAGTAACCAGGCAACA | W1 |
| **rs16944** | *IL1B* -511 | A/G | ACGTTGGATGAGAGGCTCCTGCAATTGAC  ACGTTGGATGCTGTCTGTATTGAGGGTGTG | gggaAATTGACAGAGAGCTCC | W1 |
| **rs1143634** | *IL1B* +3954 | G/A | ACGTTGGATGCATGTGCTCCACATTTCAG  ACGTTGGATGGTTCAGTGATCGTACAGGTG | caggTCAGAACCTATCTTCTT | W1 |
| **rs4251961** | *IL1RN* | T/C | ACGTTGGATGTCATTCATGCTTCCGGTGAG  ACGTTGGATGCCTTCAGACCTCATTTTGAC | AAGATAGGGCAGATAGCA | W1 |
| **rs579543** | *IL1RN* | G/A | ACGTTGGATGTCAAACCCTGACAGAACACC  ACGTTGGATGCCTTGGACCACTAAGTCTG | TCTGCTGATGGTGCC | W2 |
| **rs315952** | *IL1RN*  MspA1 11100 | T/C | ACGTTGGATGTAACATCACTGACCTGAGCG  ACGTTGGATGGCAGACTCAAAACTGGTGG | aCTTCATCCGCTCAGACAG | W1 |
| **rs2234650** | *IL1R1* Pst1 1970 | C/T | ACGTTGGATGTGAAGACTAGCGAAGTGGAG  ACGTTGGATGATCACACCTTGGGCTCCTTG | GGGGAAAGCCCGAGGGAG | W2 |
| **rs2069762** | *IL2* -330 | A/C | ACGTTGGATGCTTGTCCACCACAATATGC  ACGTTGGATGGGTGGGGATACAAAAGTAAC | accTGTTCAGTGTAGTTTTA | W2 |
| **rs2069763** | *IL2* +166 | C/A | ACGTTGGATGAACAGTGCACCTACTTCAAG  ACGTTGGATGTTCCATTCAAAATCATCTG | ccaccACTGGAGCATTTACT | W2 |
| **rs2243248** | *IL4* -1098 | T/G | ACGTTGGATGGCCCACTTTTTGAATGGAAC  ACGTTGGATGTGACTAGGAGGGCTGATTTG | TAGGAAAAAGAGCTAC | W2 |
| **rs2243250** | *IL4* -590 | C/T | ACGTTGGATGTGATACGACCTGTCCTTCTC  ACGTTGGATGTAACAGGCAGACTCTCCTAC | ggaaaTTGGGAGAACATTGT | W2 |
| **rs2070874** | *IL4* -33 | C/T | ACGTTGGATGAGACCCATTAATAGGTGTCG  ACGTTGGATGTGCATCGTTAGCTTCTCCTG | agagTTGCAGTGACAATGTGAG | W1 |
| **rs1801275** | *IL4R* +1902 | A/G | ACGTTGGATGACCCTGCTCCACCGCATGTA  ACGTTGGATGATCCTCCGCCGAAATGTCCT | CCGCATGTACAAACTCC | W1 |
| **rs1800797** | *IL6* -597 | A/G | ACGTTGGATGGAGACGCCTTGAAGTAACTG  ACGTTGGATGTCTTCTGTGTTCTGGCTCTC | tcACTGCACGAAATTTGAGG | W1 |
| **rs1800795** | *IL6* -174 | C/G | ACGTTGGATGAGCCTCAATGACGACCTAAG  ACGTTGGATGGATTGTGCAATGTGACGTCC | CTAGTTGTGTCTTGC | W1 |
| **rs2227307** | *CXCL8* (IL-8) | T/G | ACGTTGGATGACCGTGGTTCTCAATAGGAC  ACGTTGGATGGATCAATATAGATATTCTGC | ttcATATATGCATGCTAC | W2 |
| **rs4073** | *CXCL8* (IL-8) | A/T | ACGTTGGATGCTGAAGCTCCACAATTTGGT  ACGTTGGATGGTACTATATCTGTCACATGG | ctcccCAATTTGGTGAATTATCAA | W2 |
| **rs1800872** | *IL10* -592 | T/G | ACGTTGGATGAGCAGCCCTTCCATTTTAC  ACGTTGGATGAAAGGAGCCTGGAACACATC | ggaaAGAGACTGGCTTCCTACAG | W1 |
| **rs1800871** | *IL10* -819 | A/G | ACGTTGGATGGTGTACCCTTGTACAGGTG  ACGTTGGATGATGCTAGTCAGGTAGTGCTC | gtggACCCTTGTACAGGTGATGTAA | W1 |
| **rs1800896** | *IL10* -1082 | T/C | ACGTTGGATGATTCCATGGAGGCTGGATAG  ACGTTGGATGGACAACACTACTAAGGCTTC | TATCCCTACTTCCCC | W2 |
| **rs3212227** | *IL12* -1188 | T/G | ACGTTGGATGCACAATGATATCTTTGCTG  ACGTTGGATGATGGCAACTTGAGAGCTGG | CTGTATTTGTATAGTT | W2 |
| **rs2275913** | *IL17A* | G/A | ACGTTGGATGAAATTTCCGCCCCCAATGAG  ACGTTGGATGAAGAGGACATGGTCTTTAGG | GGTCATAGAAGAATCTCT | W2 |
| **rs763780** | *IL17F* | T/C | ACGTTGGATGCTTCAGCTGAGTGGATATGC  ACGTTGGATGAAGGTGCTGGTGACTGTTG | GCACCTCTTACTGCACA | W2 |
| **rs2430561** | *INFG* +874 | T/A | ACGTTGGATGCAGACATTCACAATTGATT  ACGTTGGATGGATAGTTCCAAACATGTGCG | ttccTACAACACAAAATCAAATC | W1 |
| **rs11003125** | *MBL2* -550 | G/C | ACGTTGGATGCTCAACCTTAGTCACCAACC  ACGTTGGATGGAGAAAATGCTTACCCAGGC | CCCCTTGGTGTTTTA | W2 |
| **rs1800450** | *MBL2* cd 54 | C/T | ACGTTGGATGTAGCTCTCCAGGCATCAAC  ACGTTGGATGAGAGACAGAACAGCCCAAC | AAGATGGGIGTGATG | W1 |
| **rs1800629** | *TNF* -308 | G/A | ACGTTGGATGCTGATTTGTGTGTAGGACCC  ACGTTGGATGGGAGGCAATAGGTTTTGAGG | tAGGCTGAACCCCGTCC | W1 |
| **rs361525** | *TNF* -238 | G/A | ACGTTGGATGCACACAAATCAGTCAGTGGC  ACGTTGGATGAAGCATCAAGGATACCCCTC | cctaCAGAAGACCCCCCTCGGAATC | W1 |

**Table S4. Association between functional polymorphisms in the *IL1A*, *IL1B* and *IL6* genes and infectious complications after lung transplantation.**

| **SNP**  ***Gene***  **(also known as)** | **Alleles**  **Genotypes** | **IO**  **N=41**  **[frequency]** | **FSI**  **N=62**  **[frequency]** | **OR (95% CI)** | **P-value** |
| --- | --- | --- | --- | --- | --- |
| **rs1800587**  ***IL1A***  (*IL1A* -889) | GG/AG/AA | 0.439/ 0.537/0.024 | 0.403/0.387/0.210 |  | **0.023** |
|  | AG or AA  GG | 0.561  0.439 | 0.597  0.403 | 1.16 (0.54–2.67) | 0.718 |
|  | **AA**  GG or AG | 0.024  0.976 | 0.210  0.790 | 10.6 (1.79–116) | **0.007** |
| **rs1143634**  ***IL1B***  (IL1B +3954) | GG/AG/AA | 0.561/0.415/0.024 | 0.435/0.419/0.145 |  | 0.105 |
|  | AG or AA  GG | 0.439  0.561 | 0.565  0.435 | 1.66 (0.73–3.54) | 0.212 |
|  | **AA**  GG or AG | 0.024  0.976 | 0.145  0.855 | 6.79 (1.00–76.2) | **0.043** |
| **rs16944**  ***IL1B***  (*IL1B* -511) | AA/AG/GG | 0.073/0.610/0.317 | 0.048/0.403/0.548 |  | 0.070 |
|  | AG or GG  AA | 0.927  0.073 | 0.952  0.048 | 1.55 (0.35–6.89) | 0.599 |
|  | **GG**  AA or AG | 0.317  0.683 | 0.548  0.452 | 2.62 (1.12–6.01) | **0.021** |
| **rs1800795**  ***IL6***  (*IL6* -174) | CC/CG/GG | 0.439/0.341/0.220 | 0.177/0.468/0.355 |  | **0.015** |
|  | **CG or GG**  CC | 0.561  0.439 | 0.823  0.177 | 3.63 (1.47–8.99) | **0.004** |
|  | GG  CC or CG | 0.220  0.780 | 0.355  0.645 | 1.96 (0.80–4.54) | 0.143 |
| **rs1800797**  ***IL6***  (*IL6* -597) | AA/AG/GG | 0.439/0.341/0.220 | 0.161/0.468/0.371 |  | **0.008** |
|  | **AG or GG** AA | 0.561  0.439 | 0.839  0.161 | 4.07 (1.60–9.89) | **0.002** |
|  | GG  AA or AG | 0.220  0.780 | 0.371  0.629 | 2.10 (0.87–4.86) | 0.104 |

Risk genotypes and p-‍values (χ^2^) reaching significance (p ≤ 0.05) are marked in bold. OR > 1 indicates a higher prevalence of the genotype(s) in the FSI group.

Abbreviations: CI, confidence interval; FSI, frequent and/or severe infections in inpatient settings; IO, infrequent infections managed in outpatient settings; N, sample size; OR, odds ratio; SNP, single nucleotide polymorphism.

**Table S5. Comparison of allelic frequencies in the studied single nucleotide polymorphisms in immune genes in lung transplant recipients grouped according to the frequency and severity of infections.**

| **SNP** | **Gene** | **Ref** | **Alt** | **IO**  **N=41**  **[Alt freq.]** | **FSI**  **N=62**  **[Alt freq.]** | **OR** | **95% CI** | **P-value** |
| --- | --- | --- | --- | --- | --- | --- | --- | --- |
| **rs1800587** | *IL1A* | G | A | 0.293 | 0.403 | 1.63 | 0.91–2.92 | 0.106 |
| **rs16944** | *IL1B* | A | G | 0.622 | 0.750 | 1.82 | 0.98–3.27 | **0.050** |
| **rs1143634** | *IL1B* | G | A | 0.232 | 0.355 | 1.82 | 0.96–3.37 | 0.060 |
| **rs4251961** | *IL1RN* | T | C | 0.390 | 0.379 | 0.95 | 0.55–1.68 | 0.871 |
| **rs579543** | *IL1RN* | G | A | 0.220 | 0.298 | 1.51 | 0.81–2.91 | 0.210 |
| **rs315952** | *IL1RN* | T | C | 0.317 | 0.315 | 0.99 | 0.54–1.76 | 0.969 |
| **rs2234650** | *IL1R1* | C | T | 0.366 | 0.379 | 1.06 | 0.61–1.89 | 0.848 |
| **rs2069762** | *IL2* | A | C | 0.354 | 0.274 | 0.69 | 0.37–1.28 | 0.226 |
| **rs2069763** | *IL2* | C | A | 0.195 | 0.339 | 2.11 | 1.12– 3.99 | **0.025** |
| **rs2243248** | *IL4* | T | G | 0.073 | 0.089 | 1.23 | 0.43–3.46 | 0.692 |
| **rs2243250** | *IL4* | C | T | 0.195 | 0.185 | 0.86 | 0.46–1.89 | 0.863 |
| **rs2070874** | *IL4* | C | T | 0.195 | 0.185 | 0.87 | 0.46–1.89 | 0.863 |
| **rs1801275** | *IL4R* | A | G | 0.232 | 0.226 | 0.97 | 0.49–1.88 | 0.921 |
| **rs1800797** | *IL6* | A | G | 0.390 | 0.589 | 2.24 | 1.25–3.94 | **0.005** |
| **rs1800795** | *IL6* | C | G | 0.390 | 0.605 | 2.39 | 1.34–4.23 | **0.003** |
| **rs2227307** | *CXCL8* | T | G | 0.500 | 0.532 | 1.14 | 0.64–2.01 | 0.650 |
| **rs4073** | *CXCL8* | A | T | 0.500 | 0.468 | 0.88 | 0.50–1.55 | 0.650 |
| **rs1800872** | *IL10* | T | G | 0.720 | 0.645 | 0.71 | 0.39–1.29 | 0.265 |
| **rs1800871** | *IL10* | A | G | 0.720 | 0.645 | 0.71 | 0.39–1.29 | 0.265 |
| **rs1800896** | *IL10* | T | C | 0.415 | 0.347 | 0.75 | 0.43–1.32 | 0.324 |
| **rs3212227** | *IL12* | T | G | 0.146 | 0.218 | 1.62 | 0.77–3.41 | 0.200 |
| **rs2275913** | *IL17A* | G | A | 0.354 | 0.347 | 0.97 | 0.55–1.76 | 0.919 |
| **rs763780** | *IL17F* | T | C | 0.037 | 0.032 | 0.83 | 0.23–3.56 | 0.867 |
| **rs1800629** | *TNF* | G | A | 0.159 | 0.153 | 0.96 | 0.45–2.03 | 0.918 |
| **rs361525** | *TNF* | G | A | 0.037 | 0.016 | 0.43 | 0.08–2.16 | 0.350 |
| **rs11003125** | *MBL2* | G | C | 0.354 | 0.306 | 0.81 | 0.45–1.48 | 0.479 |
| **rs1800450** | *MBL2* | C | T | 0.195 | 0.129 | 0.61 | 0.28–1.32 | 0.200 |
| **rs2430561** | *IFNG* | T | A | 0.427 | 0.492 | 1.30 | 0.74–2.24 | 0.359 |

The p-values reaching significance (p ≤ 0.05) are marked in bold.

Abbreviations: Alt, alternative allele; Alt freq, frequency of alternative allele; CI, confidence interval; FSI, frequent and/or severe infections in inpatient settings; IO, infrequent infections managed in outpatient settings; N, sample size; OR, odds ratio; Ref, reference allele; SNP, single nucleotide polymorphism.

**Table S6. Comparison of risk genotype frequencies between lung transplant recipients and the general population.**

| Genotype | Central European population^a^  N=1482 | LTRs  N=103 | | LTRs with IO  N=41 | | LTRs with FSI  N=62 | |
| --- | --- | --- | --- | --- | --- | --- | --- |
|  | Frequency | Frequency | p-value | Frequency | p-value | Frequency | p-value |
| *IL1A-889 (*rs1800587) AA | 0.082 | 0.136 | 0.060 | 0.024 | 0.179 | 0.210 | **0.001** |
| *IL1B+*3954 (rs1143634) AA | 0.053 | 0.097 | 0.076 | 0.024 | 0.394 | 0.145 | **0.003** |
| *IL1B-*511 (rs16944) GG | 0.452 | 0.456 | 0.994 | 0.317 | 0.086 | 0.548 | 0.136 |
| *IL6-*174 (rs1800795) GG/CG | 0.800 | 0.718 | 0.049 | 0.561 | **<0.001** | 0.823 | 0.657 |
| *IL6-597* (rs1800797) GG/AG | 0.806 | 0.728 | 0.057 | 0.561 | **<0.001** | 0.839 | 0.518 |

^a^ own data (unpublished). The p-values reaching significance (p ≤ 0.05) are marked in bold.

Abbreviations: FSI, frequent and/or severe infections in inpatient settings; IO, infrequent infections managed in outpatient settings; LTRs, Lung transplant recipients; N, sample size

**Table S7. Risk genotype frequencies in different world populations.**

| Population | rs1800587  (*IL1A-889* G>A)  AA freq. (N) | rs1143634  (*IL1B+*3954 G>A)  AA freq. (N) | rs16944  (*IL1B-*511 A<G)  GG freq. (N) | rs1800795  (*IL6-*174 C<G)  GG+CG freq. (N) | rs1800797  (*IL6-597* A<G)  GG+AG freq. (N) |
| --- | --- | --- | --- | --- | --- |
| European^a^ | 0.089 (318,198) | 0.057 (455,810) | 0.443 (355,650) | 0.800 (14,286) | 0.832 (351,012) |
| Central Europe^b^ | 0.082 (1,482) | 0.053 (1,482) | 0.452 (1,482) | 0.800 (1,482) | 0.806 (1,482) |
| African^a^ | 0.151 (36,708) | 0.018 (53,768) | 0.217 (46,450) | 0.995 (2,946) | 0.994 (41,164) |
| East Asian ^a^ | 0.007 (5,492) | 0.001 (7,832) | 0.279 (7,588) | 1.000 (86) | 1.000 (5,740) |
| South Asian ^a^ | 0.078 (486) | 0.030 (6,176) | 0.156 (1,282) | 1.000 (98) | 0.973 (596) |
| Latin American 1^a^ | 0.090 (6,722) | 0.035 (9,306) | 0.310 (8,490) | 0.959 (146) | 0.942 (7,692) |
| Latin American 2 ^a^ | 0.061 (15,316) | 0.016 (15,928) | 0.212 (14,202) | 0.961 (610) | 0.970 (16,050) |

^a^ ALFA project, release version: 20250407153717; ^b^ own data, unpublished

Abbreviations: N, sample size


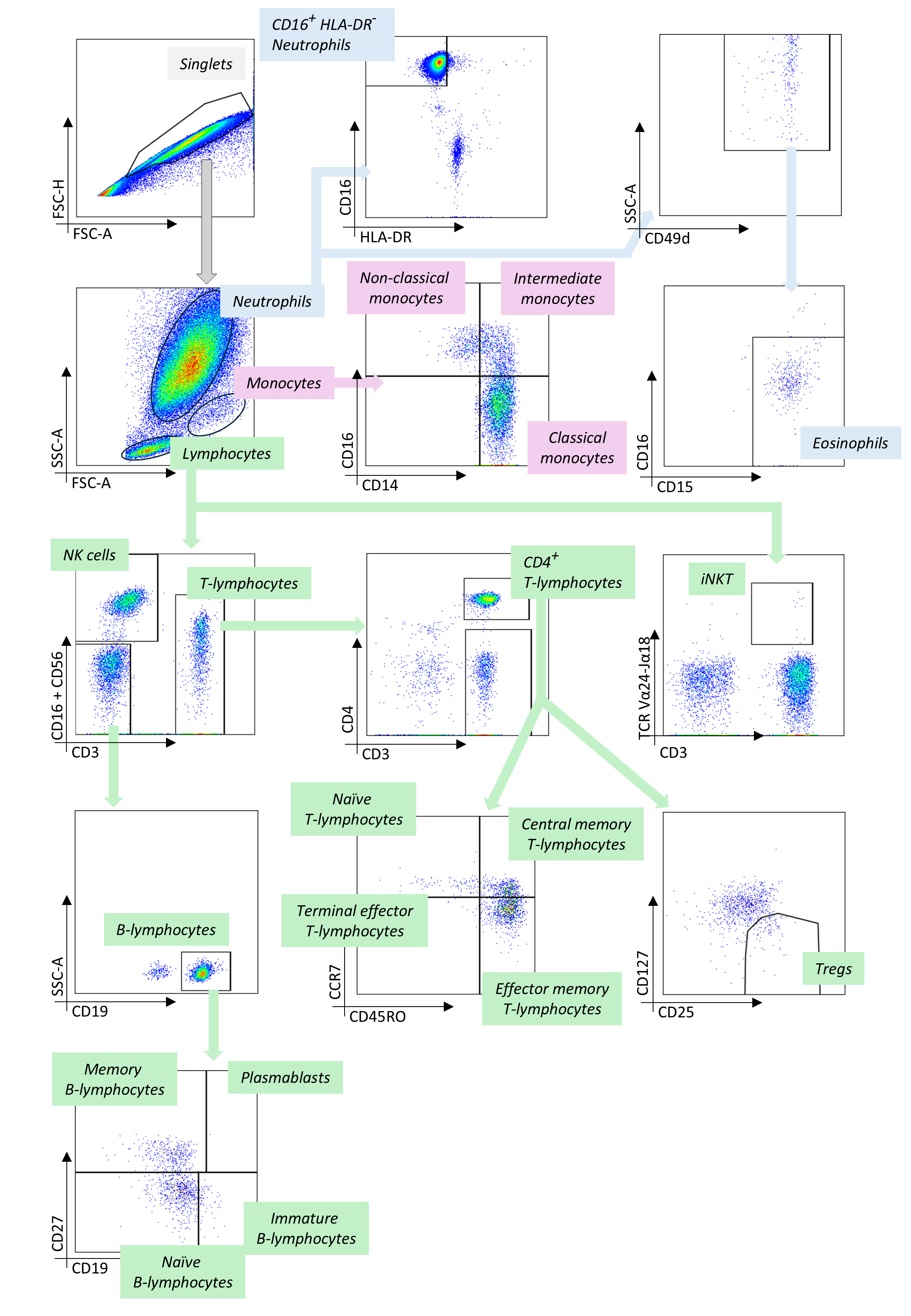


**Figure S1. Gating strategy for major populations of circulating immune cells.**

Legend: NK cells: Natural Killer cells; iNKT cells: invariant Natural Killer T cells; Tregs: regulatory T cells.


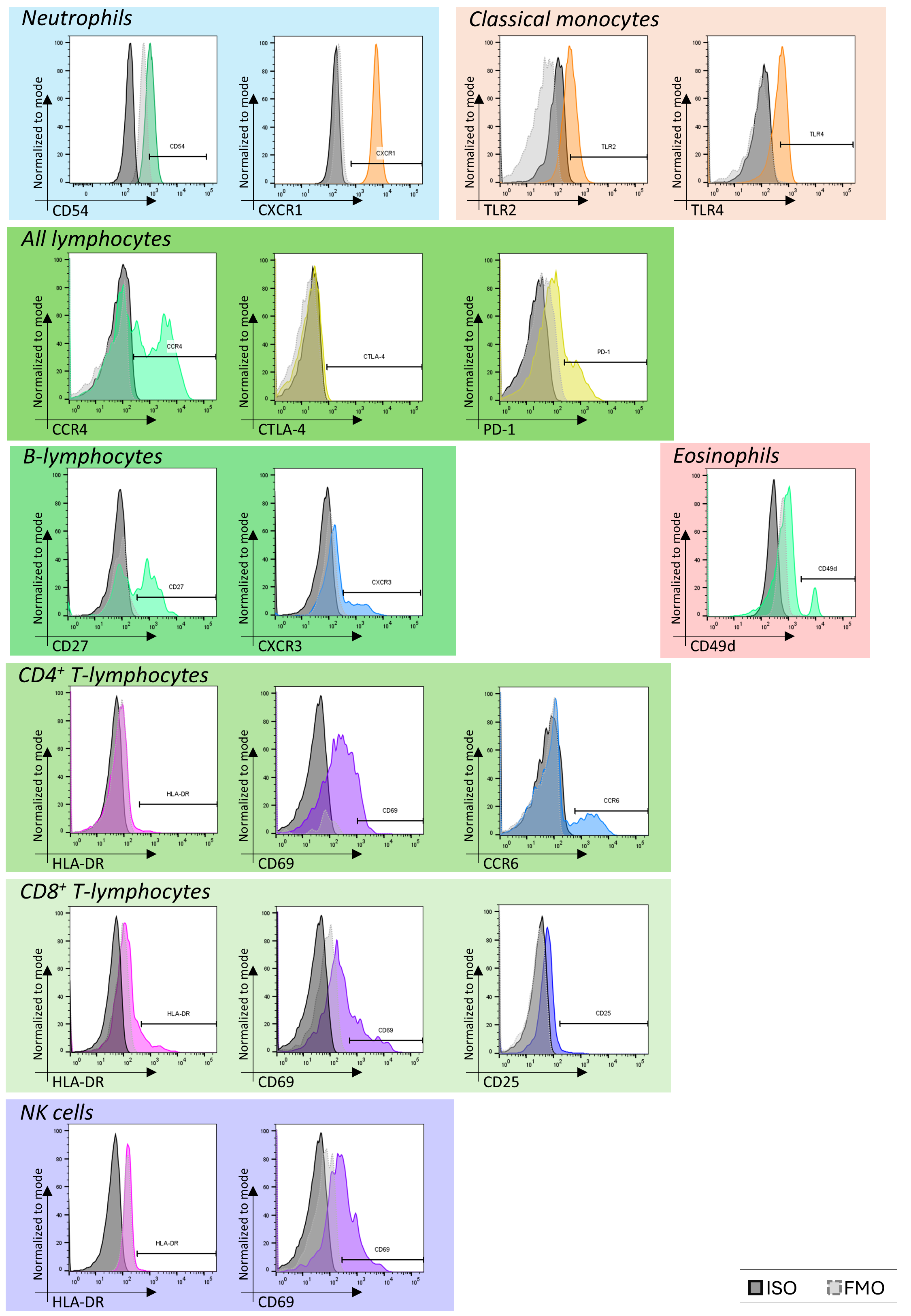


**Figure S2. Expression of analysed markers in circulating immune cells.**

Legend: NK cells: Natural Killer cells; FMO: fluorescence minus one control; ISO: isotype control.


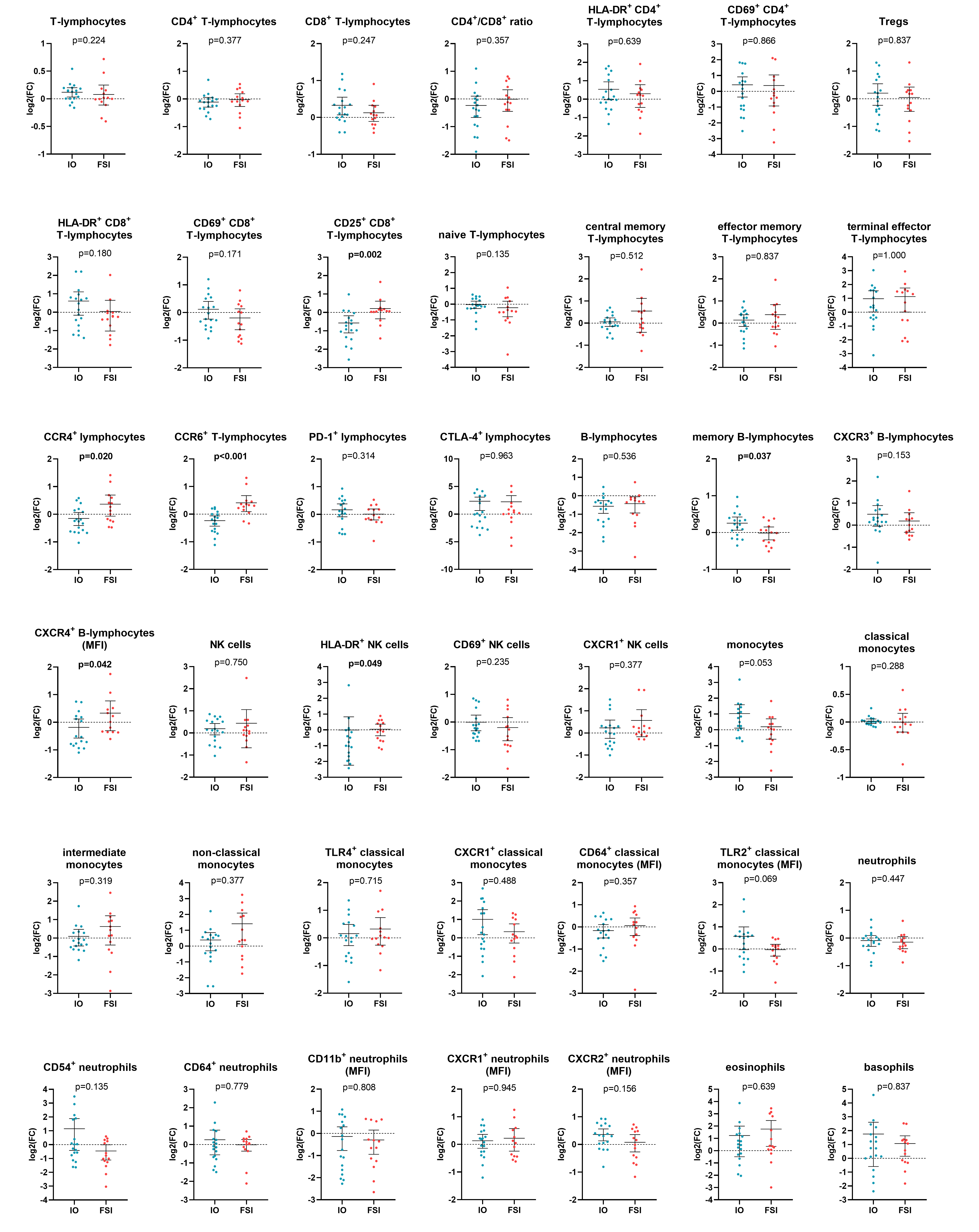


**Figure S3. Analysis of cytokine serum levels in paired samples from lung transplant recipient subgroups stratified by infection frequency and severity.**

Paired blood samples were collected at an average of 6 months and 1 year after lung transplantation (mean 5.7 months, 95% confidence interval [CI] 5.01–6.3 months, and mean 14.3 months, 95% CI 12.5–16.1 months, respectively). Patients had no signs of current infection at the time of blood collection. Differences in the proportions of immune cells in the paired samples are expressed as fold change (log_2_-scale). Patients were grouped according to the frequency and severity of infections as follows: patients with infrequent infections managed in outpatient settings (IO; n = 18), and patients frequent/severe infections in inpatient settings (FSI; n = 14). The bars represent the mean and the corresponding 95% CI.


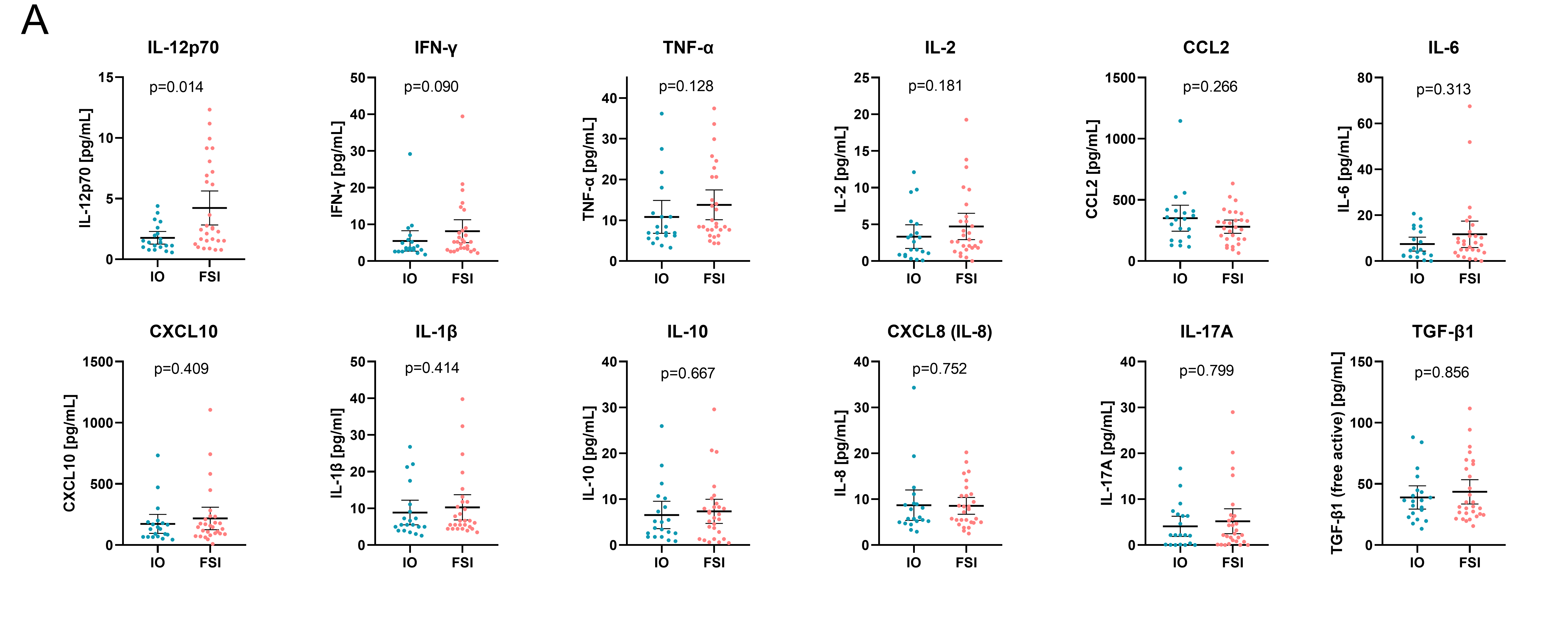


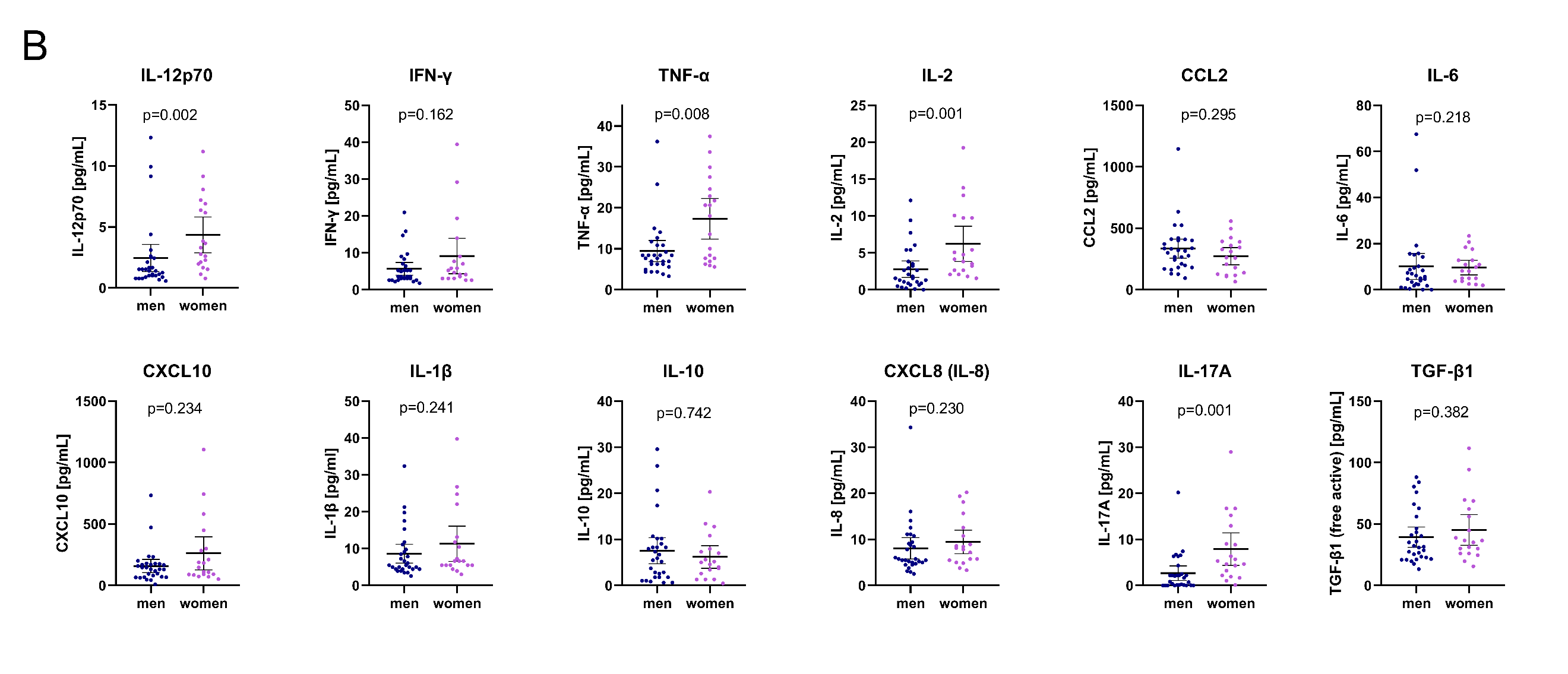


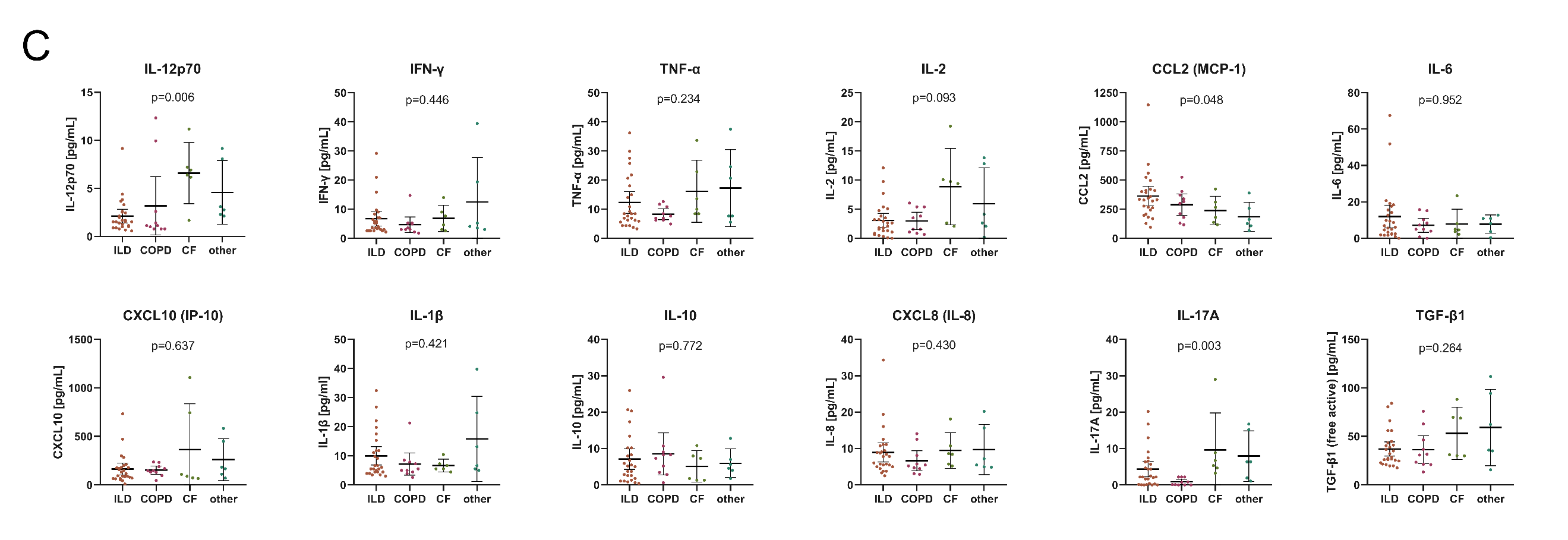


**Figure S4. Analysis of cytokine serum levels in patients after lung transplantation.**

Cytokine levels were measured in serum samples taken on average 1 year after lung transplantation (mean 13.6 months, 95% confidence interval [CI] 12.5–14.8 months). Patients had no signs of current infection at the time of blood collection. Patients were grouped according to **(A)** the frequency and severity of infections as follows: patients with infrequent infections managed in outpatient settings (IO; n = 20), and patients frequent/severe infections in inpatient settings (FSI; n = 28); **(B)** sex: 29 men, 19 women; **(C)** indication for transplantation: interstitial lung disease (ILD, n = 26), chronic obstructive pulmonary disease (COPD, n = 10), cystic fibrosis (CF, n = 6) and other conditions (n = 6). The bars represent the mean and the corresponding 95% CI; p-values ≤0.05 are considered significant.


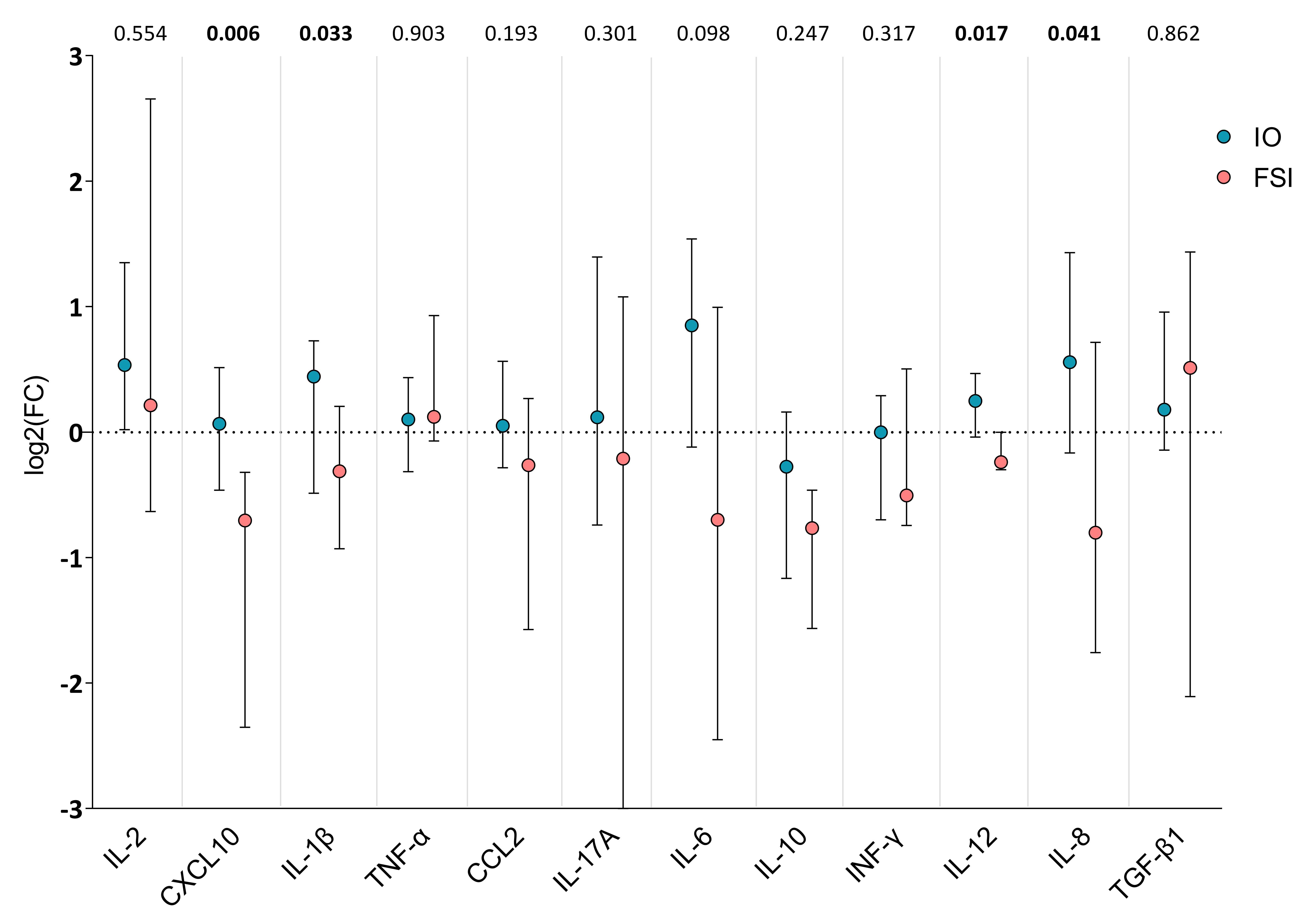
A B


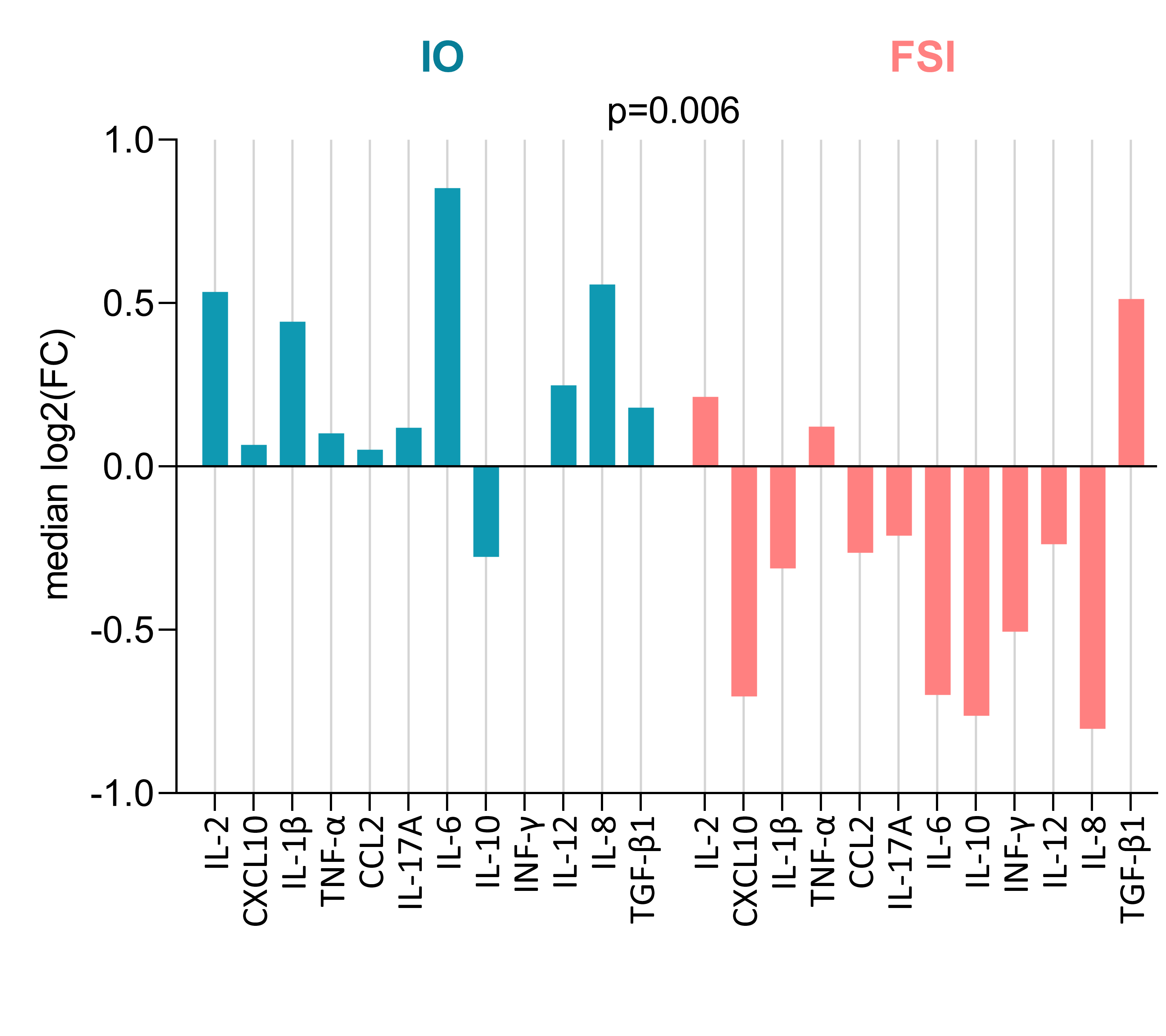


**Figure S5. Analysis of cytokine serum levels in paired samples from patients after lung transplantation.**

Cytokine levels were measured in paired serum samples taken 6 months (median; mean 6.1, 95% confidence interval [CI] 5.5–6.7 months) and 12 months (median; mean 12.9, 95% CI 11.5–14.3 months) after lung transplantation. Patients had no signs of current infection at the time of blood collection. Patients were divided according to the frequency and severity of infections as follows: patients with infrequent infections managed in outpatient settings (IO; n = 12), and patients frequent/severe infections in inpatient settings (FSI; n = 9). **(A)** Fold changes in cytokine serum levels in paired samples from patients with IO (blue) and with FSI (red). Dots represent medians and bars represent 95% CIs. P values are reported, and those reaching statistical significance (p ≤ 0.05) are highlighted in bold. **(B)** Comparison of median fold changes in cytokine levels between patients with IO and FSI. Positive values of log_2_(FC) represent an increase, negative values a decrease, and zero indicates no change in cytokine levels over time.
